# Combination of Inulin and Resistant Dextrin Has Superior Prebiotic Effects and Reduces Gas Production During In Vitro Fermentation of Fecal Samples from Older People

**DOI:** 10.3390/nu16244262

**Published:** 2024-12-10

**Authors:** Kazuma Yoshida, Eri Kokubo, Shunsuke Morita, Hirofumi Sonoki, Kazuhiro Miyaji

**Affiliations:** Health Care & Nutritional Science Institute, R&D Division, Morinaga Milk Industry Co., Ltd., 5-1-83, Higashihara, Zama 252-8583, Kanagawa, Japan; e-karita@morinagamilk.co.jp (E.K.); s-morita@morinagamilk.co.jp (S.M.); h-sonoki@morinagamilk.co.jp (H.S.); k_miyazi@morinagamilk.co.jp (K.M.)

**Keywords:** gut microbiota, prebiotics, inulin, resistant dextrin, gas production, older people, fecal fermentation

## Abstract

Background: Older people are more susceptible to deterioration of the gut microbiota. Prebiotics help improve the gut microbiota. Inulin, a major prebiotic, stimulates the growth of *Bifidobacterium*; however, it produces a large amount of gas, which leads to abdominal symptoms. Methods: In this study, in vitro fecal fermentation was performed using fecal samples from seven older people (mean subject age, 73.4 years; five men and two women) to examine whether combining inulin with another prebiotic material, resistant dextrin, could lead to decreased gas production and show prebiotic effects. Results: The *Bifidobacterium* counts and short-chain fatty acid production did not differ significantly between the inulin 0.5% group and the inulin 0.25% plus resistant dextrin 0.25% combination group. However, the inulin 0.25% plus resistant dextrin 0.25% combination group had lower gas production than the inulin 0.5% group (*p* < 0.10). Furthermore, compared with the inulin 0.5% group, the 0.25% combination group showed significantly greater gut microbiota diversity and tended toward a lower pH in the fermentation medium at the end of fermentation (*p* = 0.09). These effects are believed to be due to the combination of inulin, which is highly selective for *Bifidobacterium* and rapidly utilized by the gut microbiota, and resistant dextrin, which is slowly utilized by various bacterial genera. Conclusions: These findings suggest that the inulin plus resistant dextrin combination has superior prebiotic effects in older people and causes less gas production than inulin alone.

## 1. Introduction

The human gut microbiota deteriorates with aging, leading to a decrease in the abundance of beneficial bacteria, including the *Bifidobacterium* genus [1,2]. Gut microbiota deterioration has been found to impair intestinal function and the immune system [3,4]; additionally, it is associated with diseases such as diabetes and kidney disease [5]. Therefore, the improvement of the gut microbiota is particularly crucial for older individuals.

A prebiotic is defined as “a substrate that is selectively utilized by host microorganisms conferring a health benefit”; prebiotics include nondigestible carbohydrates, such as fibers and oligosaccharides, that help improve the gut microbiota [6]. The ability of the gut microbiota to access non-digestible carbohydrates varies depending on several factors, including their chain length, molecular weight, and chemical structure [7]. In general, oligosaccharides are rapidly utilized by the gut microbiota owing to their short chain lengths and low molecular weights. In contrast, soluble dietary fiber, which has a long chain length and high molecular weight, is slowly utilized by the gut microbiota.

Inulin, one of the major prebiotics, is a type of fructan composed of β-(2, 1) fructosyl-fructose bonds. It has a linear structure. Although inulin is a soluble dietary fiber, its structure is similar to that of oligosaccharides, and it can be easily utilized by the gut microbiota [8]. Inulin is highly selective for *Bifidobacterium* and reportedly exhibits prebiotic effects, including promoting *Bifidobacterium* growth (bifidogenic effect) and short-chain fatty acid (SCFA) production [9,10]. However, inulin is rapidly utilized in large quantities owing to its easy utilization by the gut microbiota, leading to significant gas production in the intestine, which can cause abdominal symptoms such as bloating [11,12]. In addition, inulin cannot exert a prebiotic effect in the distal colon as it is primarily utilized in the proximal colon and does not reach the distal colon [13].

High gas production from inulin can be mitigated by combining it with other prebiotics. For example, studies on in vitro human fecal fermentation have demonstrated that combining inulin with acacia gum, which is slowly utilized by the gut microbiota, leads to bifidogenic effects and decreased gas production [13]. Combining other slowly utilized prebiotic materials with inulin may yield similar effects. However, the utilization of prebiotic materials by the gut microbiota depends on their composition and structure. Therefore, it is necessary to determine if combining inulin with these materials can produce similar effects.

Resistant dextrin is a soluble fiber synthesized from starch found in wheat and maize. Similar to normal dextrin, resistant dextrin is characterized by its 1→4 glucoside linkages. In addition to its 1→6 linkages, resistant dextrin also has several branched structures, such as 1→2 and 1→3 linkages [14]. Resistant dextrin slows the rise in blood glucose and triglyceride levels after meals, and it is widely used in health-food products in Japan [15,16]. Resistant dextrin is utilized more slowly and produces less gas than inulin [17]. Therefore, a resistant dextrin plus inulin combination may exert prebiotic effects and cause lesser gas production than inulin alone. This could be used to develop health-food products for older people. However, this aspect has not yet been investigated in detail.

In vitro fecal fermentation assays are conducted to study the effects of prebiotics on the stimulation of the growth of beneficial bacteria and the production of useful metabolites [7]. This approach helps verify the prebiotic effects by measuring both the microbiota and metabolite levels in the fermentation medium. Additionally, when prebiotics are utilized, useful metabolites, such as SCFAs, are produced and the pH of the medium decreases, thus mimicking the conditions in the human intestinal tract [18]. Therefore, the prebiotic utilization rate can be determined by measuring the fermentation medium pH [13]. Furthermore, the amount of gas produced by the gut microbiota can be measured by examining the gas production in the fermentation vessel [17,19]. Although examining these effects in human studies is challenging, they can be easily verified using in vitro fecal fermentation.

The selection of individuals is crucial in fecal fermentation studies because gut microbiota characteristics can vary among individuals. The response to prebiotics has been found to differ between older and younger individuals because of age-related changes in the gut microbiota [20,21]. Studies comparing the effects of prebiotics on older people mainly focus on single prebiotic materials [22,23,24]. To the best of our knowledge, no studies have compared the gas production and prebiotic effects of prebiotic combinations with those of each material alone in older people. Therefore, in this study, we specifically used fecal samples from older individuals to investigate the effects of prebiotics in this age group.

The objective of this study was to examine whether using a combination of inulin and resistant dextrin would provide prebiotic effects, such as bifidogenic effects and increased SCFA production, and whether this combination would cause lesser gas production than inulin alone. Therefore, we performed an in vitro fecal fermentation study with fecal samples from older people.

## 2. Materials and Methods

### 2.1. Sample Collection

We collected fecal samples from healthy older participants in Japan. Participants who were suspected of having an infectious bowel disease were excluded to prevent infection during fermentation. Participants were recruited through a clinical trial recruitment site https://www.seikatsu-kojo.jp/ (accessed on 8 December 2024). To recruit participants, the exam details were posted on the recruiting site by the agency operating this site. Thirteen participants were recruited from the pool of healthy older Japanese individuals who were registered on the recruiting site. The participants collected the fecal samples in small stool tubes and stored them under anaerobic conditions using an AnaeroPouch (Mitsubishi Gas Chemical, Tokyo, Japan), which was then kept in the household freezer. To limit the burden on the participants, we requested that each participant provide only one fecal sample. Within 48 h of collection, the frozen fecal samples were shipped with dry ice to the Morinaga Milk Industry laboratory and stored at −80 °C.

Five participants were excluded from the study due to improper sample collection methods, such as the failure to maintain anaerobic conditions. Additionally, one sample did not show *Bifidobacterium* and was therefore not used in this study, which focused on *Bifidobacterium* growth. Finally, seven samples were used for fermentation (mean subject age, 73.4 years; age range, 72–75 years; five men and two women). Written informed consents were obtained from the participants before the start of the study. The study protocol was approved by the Ethics Committee of the Japan Conference of Clinical Research (Tokyo, Japan; protocol code: NFSFA-01; date of approval: 18 October 2019).

### 2.2. In Vitro Fecal Fermentation

Fecal fermentation was performed using a pH-controlled multichannel jar fermenter (Bio Jr. 8; ABLE, Tokyo, Japan), following the methods described in a previous study [22]. A carbon source was added to yeast extract, casitone, and fatty acid (YCFA) medium (Supplementary Table S1), and 100 mL of medium was filled in each vessel. The fermentation process was performed under anaerobic conditions (100% CO_2_) at a temperature of 37 °C, with constant stirring at 200 rpm and the pH maintained above 6.0. Inulin (Fuji FF; Fuji Nihon Seito Co., Tokyo, Japan), resistant dextrin (Fibersol-2; Matsutani Chemical Industry Co., Ltd., Hyogo, Japan), and a mixture of both were used as carbon sources. In the single carbon source groups, inulin (Inu0.5% group) or resistant dextrin (RD0.5% group) was added at a concentration of 0.5%. In the combination groups, a mixture containing 0.25% of each carbon source (Inu0.25%RD0.25% group) or 0.5% of each carbon source (Inu0.5%RD0.5% group) was used.

The frozen stored fecal samples were thawed and diluted with saline to a concentration of 10%. A volume of 100 μL of diluted fecal samples was added to each vessel. A 100 μL sample of the fecal dilution was used for bacterial community analysis. After 4 h of fermentation, the CO_2_ supply was stopped, and a gas collection bag (Laboratory for Expiration Biochemistry Nourishment Metabolism Co., Ltd., Nara, Japan) was connected to each vessel. The exhaust port was sealed with a clip, and the vessel was sealed for gas measurement. The dissolved oxygen concentration was monitored during fermentation to ensure that anaerobic conditions were maintained. If the dissolved oxygen concentration exceeded 0.5%, indicating a failure to maintain anaerobic conditions, fecal fermentation was repeated. The medium’s pH was measured every 30 min for 24 h; the pH value at 23.5 h was used as the 24 h value for data analysis. After 24 h of fermentation, the gas collection bags were removed, and the medium was collected for SCFA and gut microbiota analyses. The amount of gas accumulated in the bag was measured using a syringe. Centrifugation of the diluted fecal sample remainder and the medium after 24 h of fermentation was performed at 8000× *g* for 3 min at 4 °C. The sediment was collected for bacterial analysis, and the supernatant was used for SCFA measurements and thin-layer chromatography (TLC). Fecal fermentation was performed once for each fecal sample.

### 2.3. Microbiota Analysis

The bead-beating method was used to extract the total DNA. Following extraction, the V3–V4 region of the bacterial 16S rRNA gene was amplified and sequenced according to a previously described method [25].

### 2.4. RT-PCR

RT-PCR was performed using a QuantStudio3^®^ RT-PCR system (Thermo Fisher Scientific, Waltham, MA, USA) and TB Green^®^ Premix Ex Taq™ Tli RNaseH Plus (TaKaRa Bio Inc., Shiga, Japan). The primers F: 5′-CTCCTGGAAACGGGTGG-3′ and R: 5′-GGTGTTCTTCCCGATATCTACA-3′ were used to determine the total *Bifidobacterium* count, as previously described [26]. To determine the total bacterial count, the primers F: 5′-CCTACGGGRSGCAGCAG-3′ and R: 5′-ATTACCGCGGCTGCT-3′ were used, as previously described [27]. The amplification process was conducted as follows: it commenced with an initial hold at 95 °C for 10 s, followed by 40 cycles each at 95 °C for 3 s, 55 °C for 20 s, and 72 °C for 30 s. Fluorescent products were detected at the end of each cycle, and melting curves were generated from 60 °C to 95 °C, with increments of 0.1 °C/s. All the samples were evaluated in duplicate. The detection limit was set at 10^5^ copies/mL.

### 2.5. SCFA Analysis

SCFAs (acetic acid, propionic acid, and butyric acid) were analyzed via liquid chromatography performed using a Shimadzu Organic Acid Analysis System (Shimadzu Co., Kyoto, Japan). The culture supernatants were diluted 10 times and filtered through a 0.22 μm membrane filter (TORAST disc nylon membrane; Shimadzu Co.) before analysis. The HPLC system consisted of three Shim-pack Fast-OA columns (size, 100 mm × 7.8 mm internal diameter [ID]) connected in series, along with a Shim-pack Fast-OA guard column (size, 10 mm × 4.0 mm ID). The eluent used was 5 mmol/L *p*-toluenesulfonic acid, and the reaction solution comprised 5 mmol/L *p*-toluenesulfonic acid, 100 μmol/L ethylenediaminetetraacetic acid, and 20 mmol/L Bis-Tris (Shimadzu Co.). The flow rate and oven temperature were set at 0.8 mL/min and 50 °C, respectively. The conductivity detector CDD-10AVP (Shimadzu) was used for the measurements.

### 2.6. TLC Analysis

TLC was performed to measure the resistant dextrin and inulin residues remaining in the medium’s supernatant at the start of incubation and end of fermentation, following a previously described method [25,28]. The samples were spotted onto precoated silica gel 60 TLC aluminum plates (Merck, Darmstadt, Germany) and developed using a solvent composed of ethyl acetate, acetic acid, 2-propanol, formic acid, and water in a ratio of 25:10:5:1:15. The plates were then treated with a reagent comprising diphenylamine, aniline, and phosphoric acid. This reagent was prepared by blending 100 mL acetone (FUJIFILM Wako Pure Chemical Corp., Osaka, Japan), 1 g diphenylamine (Nacalai Tesque Inc., Kyoto, Japan), 1 mL aniline (FUJIFILM Wako Pure Chemical Corp.), and 10 mL phosphoric acid (Hayashi Pure Chemical Industry, Ltd., Osaka, Japan). After development, the samples were visualized by heating in an oven.

### 2.7. Statistical Analysis

Statistical analysis was performed using JMP version 13.2.1 (SAS Institute, Cary, NC, USA). The gas production and the Shannon index were compared using the Steel–Dwass test. The bacterial counts, SCFAs, and pH were compared using the Tukey–Kramer multiple comparison test. The correlation between the gas production and the bacterial population was calculated using Spearman’s rank correlation coefficient. Statistical significance was set at *p* < 0.05.

## 3. Results

### 3.1. Gas Production

**Figure 1:**
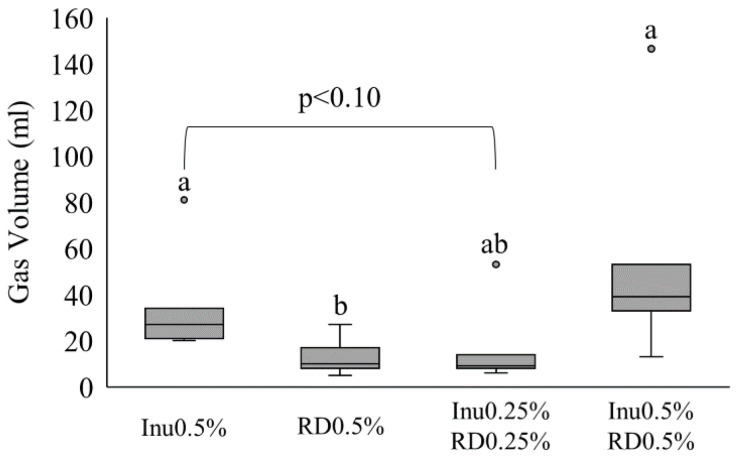
Gas production during 4–24 h of fermentation. Different letters indicate significant differences (*p* < 0.05, Steel–Dwass test).

The gas production in the Inu0.5% group and Inu0.5%RD0.5% combination group was significantly higher than that in the RD0.5% group (*p* < 0.05, Figure 1). Additionally, the gas production in the Inu0.25%RD0.25% combination group was lower than that in the Inu0.5% group (*p* < 0.10).

### 3.2. Microbiota Composition

**Figure 2:**
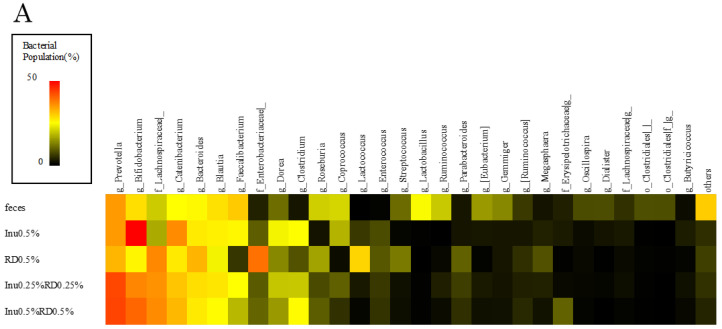

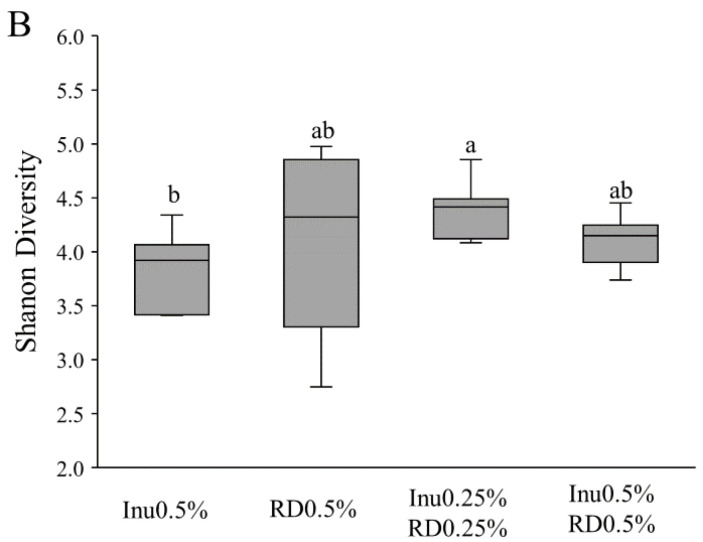
Bacterial population and microbiota diversity after 24 h of fermentation. (**A**) Top 30 predominant bacterial genera in the feces and fecal fermentation samples. Colors indicate the percentage of each bacterial population. (**B**) Box plot showing the α-diversity measured using the Shannon index after 24 h of fermentation. Different letters indicate significant differences (*p* < 0.05, Steel–Dwass test).

The bacterial composition differed among the groups (Figure 2A). The proportion of *Bifidobacterium* was highest in the Inu0.5% group (26.1%), followed by that in the Inu0.5%RD0.5% combination group (17.7%), the Inu 0.25%RD0.25% combination group (15.1%), and the RD0.5% group (5.8%). Additionally, the *Prevotella* genus proportion was higher in the combination groups than in the prebiotic alone groups. The microbiota diversity (α-diversity: Shannon index) was the lowest in the Inu0.5% group; the diversity in the Inu0.25%RD0.25% combination group was significantly higher than that in the Inu0.5% group (*p* < 0.05, Figure 2B).

The correlation between the bacterial genera and the gas production was examined to investigate the bacteria involved in gas production. A significant correlation was observed only for the *Clostridium* genus (R = 0.58). However, this genus was detected in only one sample, and no significant correlation was observed with the other bacterial genera (Supplementary Table S2A,B).

### 3.3. Bifidobacterium and Total Bacterial Counts

The *Bifidobacterium* and total bacterial counts were determined using RT-PCR. In all the inulin-containing groups (Inu0.5%, Inu0.25%RD0.25%, and Inu0.5%RD0.5%), the *Bifidobacterium* count was significantly higher than that in the RD0.5% group (Table 1). The total bacterial count was the highest in the Inu0.5%RD0.5% combination group, followed by the Inu0.5% group and the Inu0.25%RD0.25% combination group, which had almost equal counts, and the RD0.5% group. Notably, the total bacterial count was significantly higher in the Inu0.5%RD0.5% group than in the RD0.5% group.

### 3.4. SCFA Production

The acetate level in all the inulin-containing groups was significantly higher than that in the RD0.5% group (Table 2). The propionate and butyrate levels did not significantly differ between the groups, but all the inulin-containing groups had higher levels than the RD0.5% group. Additionally, the total acetate, propionate, and butyrate levels in all the inulin groups were significantly higher than that in the RD0.5% group; furthermore, the Inu0.5%RD0.5% combination group showed a significantly higher total level than the Inu0.25%RD0.25% combination group.

### 3.5. pH

**Figure 3:**
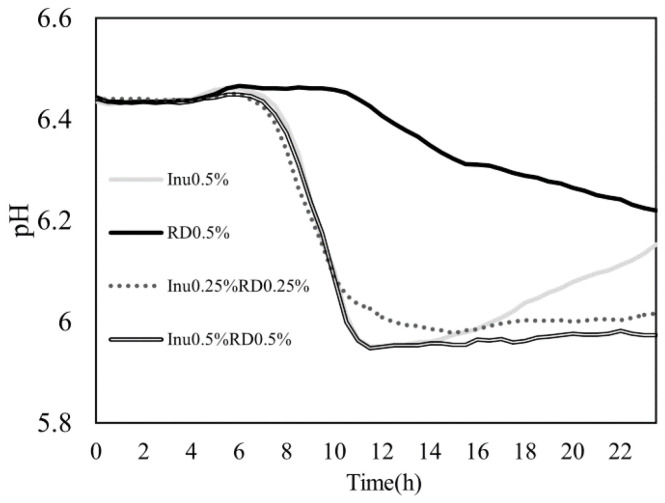
Changes in the pH of the fermentation medium during the 24 h fermentation process. Values are presented in terms of the mean (*n* = 7). Inu0.5%, RD0.5%, RD0.25%Inu0.25%, and RD0.5%Inu0.5% are depicted using a light gray line, a black line, a dotted line, and double lines, respectively.

The medium’s pH in the Inu0.5% group increased after 14 h of fermentation (Figure 3). After 24 h of fermentation, it was higher (*p* = 0.09) than that in the Inu0.25%RD0.25% combination group and significantly higher than that in the Inu0.5%RD0.5% combination group (Table 3). The RD0.5% group showed a slower decrease than all the inulin groups, with the pH being significantly higher than that of the other groups at 12 h after fermentation.

### 3.6. Inulin and Resistant Dextrin Residues in the Culture Supernatant

**Figure 4:**
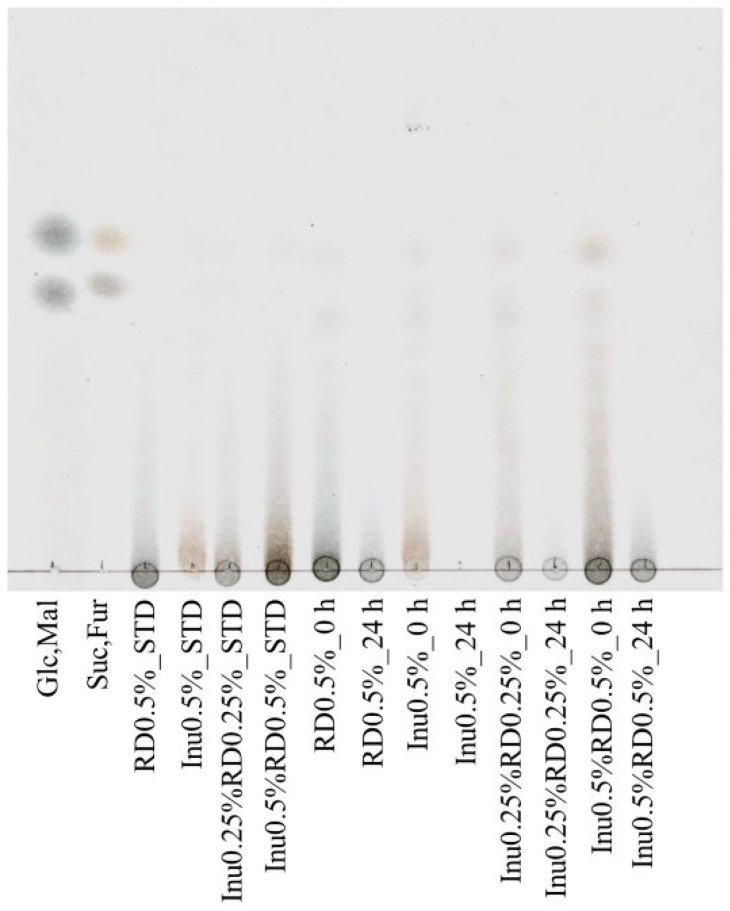
Thin-layer chromatography (TLC) showing the resistant dextrin and inulin degradation in the fecal fermentation supernatants at 0 (0 h) and 24 (24 h) h after fermentation. A representative sample image is shown. Glucose (Glc), maltose (Mal), sucrose (Suc), and fructose (Fur) spots are size markers. The standard (STD) was prepared by dissolving each prebiotic in distilled water.

The inulin and resistant dextrin residues in the culture supernatant after fermentation were analyzed using TLC (Figure 4). Inulin spots were not detected for the supernatant after fermentation, indicating that all of the inulin had been used by the fecal microbiota. In contrast, resistant dextrin spots remained after fermentation, confirming that the resistant dextrin was retained after 24 h of fermentation.

## 4. Discussion

In this study, in vitro fermentation was performed using fecal samples obtained from older individuals. The results suggest a combination of inulin and resistant dextrin can elicit prebiotic effects, including a bifidogenic response and SCFA production. Moreover, gas production was also reduced when compared to using inulin alone. Additionally, we found that the inulin plus resistant dextrin combination increased the diversity of the gut microbiota and helped to maintain a low pH in the culture medium. These results suggest that the combination of inulin and resistant dextrin may have superior prebiotic effects than each prebiotic material alone. To the best of our knowledge, this study not only examines the prebiotic effects of inulin and resistant dextrin combinations but also provides valuable insights as it utilizes fecal samples from older people.

The Inu0.25%RD0.25% combination group showed lesser gas production than the Inu0.5% group. This finding suggests that reducing the total inulin level leads to a decrease in gas production, consistent with the findings of a previous study that showed that replacing some of the inulin with other prebiotics reduced gas production [13]. Human studies have also shown that replacing some of the fructo-oligosaccharide (a high gas-producing material) with acacia gum (a slowly fermented material) can lead to reduced gas production and alleviate abdominal symptoms, such as belching [29]. These findings suggest that, if the total intake of prebiotics is the same, an inulin and resistant dextrin combination would be more effective in reducing abdominal symptoms than inulin alone.

In this study, we examined the Inu0.5%RD0.5% combination group to assess whether gas production can be additively increased when inulin is combined with resistant dextrin. As we expected, the Inu0.5%RD0.5% combination group showed more gas production than the Inu0.5% group, suggesting an additive increase in gas production due to the combination with resistant dextrin. However, the Inu0.25%RD0.25% combination group, which had half the amount of inulin and resistant dextrin compared to the Inu0.5%RD0.5% combination group, produced less than half the amount of gas as that group. Thus, the findings suggest that gas production is not necessarily related to the amount of prebiotics added, and there might be a certain threshold for the prebiotic quantity and blending ratio that leads to gas production. Previous human studies have reported that inulin and fructo-oligosaccharide do not cause abdominal symptoms when administered at low doses; however, they can cause abdominal symptoms once the intake exceeds a certain threshold [11,30]. An in vitro study that assessed the prebiotic effects of an inulin and polydextrose mixture in two different ratio combinations (inulin:polydextrose = 1:1 or 1:2), found that the amount of gas production was the same regardless of the combination ratios [31]. Therefore, examining different amounts and blending ratios of inulin and resistant dextrin combinations could clarify the amounts and blending ratios that do not cause gas production.

*Bifidobacterium* is a beneficial taxon that maintains intestinal and immune function [32]. *Bifidobacterium* counts decline with age and are also reduced in sarcopenia [1,33]. Therefore, increasing the *Bifidobacterium* counts in older people is important. Inulin is highly selective for *Bifidobacterium*; some studies have suggested that the combination of inulin with other prebiotics, which are less selective for *Bifidobacterium*, can potentially enhance or maintain a bifidogenic effect [19,31]. In the current study, although the Inu0.25%RD0.25% combination group showed a slight decrease in the *Bifidobacterium* count and proportion compared to the Inu0.5% group, these values were significantly higher than those in the RD0.5% group. A recent human study has also demonstrated an increased *Bifidobacterium* count when an inulin plus resistant dextrin combination was used [34]. Therefore, replacing some of the inulin with resistant dextrin could help increase or maintain the bifidogenic effects, with less gas production than when administering inulin alone.

The SCFAs, particularly acetate, propionate, and butyrate, produced by the gut microbiota from prebiotics are important for host energy regulation and immunomodulation; however, their production is reduced in older people [35,36]. In the current study, the Inu0.25%RD0.25% combination group showed a slightly lower acetate level than the Inu0.5% group but a significantly higher level than the RD0.5% group. Acetate is a beneficial metabolite that promotes glucagon-like peptide 1 secretion, thereby increasing energy expenditure and fat oxidation [37]. *Bifidobacterium* is an acetate-producing bacterial genus [38]. The Inu0.25%RD0.25% combination group also demonstrated an increase in the *Bifidobacterium* counts, explaining the increased acetate levels compared to those of the RD0.5% group. Additionally, the Inu0.5%RD0.5% combination group had a higher acetate level than the Inu0.5% group. These findings suggest that *Bifidobacterium* rapidly grows by utilizing inulin and that the growing *Bifidobacterium* utilizes resistant dextrin, resulting in increased acetic acid production. Propionate and butyrate were also elevated in the Inu0.5% group and combination groups compared to the RD0.5% group, although the differences were not statistically significant. These results suggest that using an inulin plus resistant dextrin combination leads to an increase in SCFA production.

This study showed that the inulin plus resistant dextrin combination had superior prebiotic effects than inulin alone, that is, it increased the microbiota diversity and decreased the fermentation medium’s pH. A decrease in the gut microbiota diversity is associated with various diseases, such as obesity [39,40]. It is suggested that prebiotic intake is effective in maintaining this diversity [41]. In the current study, the Inu0.25%RD0.25% combination group had greater microbiota diversity than the Inu0.5% group. The utilization of prebiotics by different bacteria depends on the prebiotic structure; prebiotics with more complex structures may be utilized by a greater variety of bacteria, leading to an increase in the microbiota diversity [42]. Increased diversity was also observed in another study that investigated the effects of inulin combined with pectin, which has a more complex structure than inulin [43]. Furthermore, an in vivo study showed that a combination of galacto-oligosaccharides and inulin, which are highly selective for *Bifidobacterium*, with dietary fiber, increased various bacterial species owing to their low selectivity, resulting in increased microbiota diversity [44]. Resistant dextrin is utilized by various bacterial species and has been reported to particularly increase the abundance of the *Fusicatenibacter* genus [45]. In this study, the resistant dextrin plus inulin combination may also increase the diversity, suggesting that combinations of inulin with other soluble fibers with low selectivity may increase the microbiota diversity compared to using inulin alone.

An increased intestinal pH inhibits the growth of beneficial bacteria, such as butyrate-producing bacteria, and increases the number of bacteria producing putrefactive products harmful to the host [18,46]. The decrease in the intestinal pH is induced by SCFAs derived from prebiotics [18]. Consistent with the results of a previous study, the pH decreased faster in the inulin-only group than in the resistant-dextrin-only group [17]. However, during fermentation, the pH of the inulin-only group gradually increased and was higher than that of the resistant-dextrin-only group at the end of fermentation. Inulin residue was not detected in the supernatant after fermentation, suggesting that all the inulin had been utilized by the gut microbiota during fermentation. Therefore, although the amount of SCFAs at 24 h was higher in the inulin-only group than in the resistant-dextrin-only group, the production of SCFAs from inulin decreased later in the fermentation process and the lower pH could not be maintained. Similar results were observed in a fecal fermentation study comparing the prebiotic effects of several arabinoxylan materials. Some materials increased the pH at the end of fermentation, suggesting that the materials may have been utilized completely [47]. Even in vivo, in the distal colon, where prebiotics are less accessible, the production of SCFAs by the gut microbiota is limited, resulting in a higher pH than that of the proximal colon [48]. In contrast, when inulin was combined with resistant dextrin, a lower pH was maintained even after 24 h of fermentation. The presence of resistant dextrin residue in the culture supernatant after fermentation suggested that SCFA production continued during fermentation. Additionally, no increase in the pH was observed in the combination groups, which may be due to the utilization of resistant dextrin after inulin consumption by the gut microbiota. When prebiotics with different fermentation speeds are combined, the faster material is utilized first, then the slower material is utilized, suggesting the prebiotic effect is sustained for longer [49,50]. These results suggest that the inulin plus resistant dextrin combination was effective in lowering the intestinal pH and maintaining the acidic condition.

The main limitation of this study is its small sample size. Therefore, these findings may not be generalizable to other older populations. However, our study provides valuable insights, despite the small sample size, owing to its analysis of fecal samples from older people. A previous in vitro fecal fermentation study had reported that middle-aged individuals produced more gas from prebiotics than young adults; this finding suggests that abdominal symptoms are more likely to occur with an increase in age [21]. Although the current study did not directly compare fecal samples from older people with those from younger people, it showed that the inulin plus resistant dextrin combination may have prebiotic effects in older people, with lesser gas production than that with inulin alone. In addition, this study provides limited mechanistic insights into how the combination of inulin and resistant dextrin leads to the observed effects. Future studies should compare samples from people of different age groups and health statuses, and examine the prebiotic dose-dependence, to provide further insights into the mechanisms. Finally, this in vitro study administrated prebiotics only once, so it is unclear whether similar effects would be observed with long-term intake in humans. Further clinical trials are required to confirm whether this composition exhibits prebiotic effects while reducing abdominal symptoms in older people.

## 5. Conclusions

The inulin plus resistant dextrin combination showed prebiotic effects, such as increased *Bifidobacterium* counts and SCFA production, and lesser gas production, than inulin alone. Additionally, it showed potential benefits, such as an increase in the microbiota diversity and maintenance of a lower pH. These effects are believed to be attributable to the combination of prebiotic materials that are highly selective for *Bifidobacterium* and are rapidly utilized by the gut microbiota along with prebiotic materials that are slowly utilized by various bacterial genera. These findings can be applied to the development of food products combining inulin with resistant dextrin for older people, which would provide a prebiotic that also addresses the issue of abdominal symptoms.

## Figures and Tables

**Table 1:** *Bifidobacterium* and total bacterial cell counts after 24 h in the fecal fermentation sample, determined using RT-PCR.

	Total_Bifidbacterium		Total_Bacteria	
	Cell Number (/mL)		Cell Number (/mL)	
	Mean	SD	Mean	SD
Inu0.5%	9.64 ^a^	0.26	10.06 ^ab^	0.16
RD0.5%	8.44 ^b^	0.33	9.86 ^b^	0.41
Inu0.25%RD0.25%	9.45 ^a^	0.22	10.05 ^ab^	0.17
Inu0.5%RD0.5%	9.69 ^a^	0.27	10.26 ^a^	0.17

Different letters indicate significant differences (*p* < 0.05, Tukey–Kramer multiple comparison test).

**Table 2:** Short-chain fatty acids in the medium after 24 h of fecal fermentation, measured using HPLC.

	Acetic Acid (mM)		Propionic Acid (mM)		Butyric Acid (mM)		Acetic + Propionic + Butyric Acid (mM)	
	Mean	SD	Mean	SD	Mean	SD	Mean	SD
Inu0.5%	87.3 ^ab^	7.5	16.1	6.6	6.0	4.5	109.3 ^ab^	15.2
RD0.5%	58.6 ^c^	14.4	12.4	3.9	2.9	3.6	73.9 ^c^	18.6
Inu0.25% RD0.25%	77.7 ^b^	5.5	14.7	6.0	5.5	2.9	97.9 ^b^	10.8
Inu0.5% RD0.5%	100.2 ^a^	6.8	14.3	8.6	5.6	4.8	120.0 ^a^	12.0

Different letters indicate significant differences (*p* < 0.05, Tukey–Kramer multiple comparison test).

**Table 3:** pH after 0, 12, and 24 h of fermentation.

	pH_0 h		pH_12 h		pH_24 h	
	Mean	SD	Mean	SD	Mean	SD
Inu0.5%	6.44	0.02	5.95 ^b^	0.01	6.15 ^ab^	0.09
RD0.5%	6.44	0.01	6.41 ^a^	0.06	6.22 ^a^	0.17
Inu0.25%RD0.25%	6.44	0.01	6.01 ^b^	0.06	6.02 ^bc^	0.05
Inu0.5%RD0.5%	6.44	0.01	5.95 ^b^	0.01	5.97 ^c^	0.05

Different letters indicate significant differences (*p* < 0.05, Tukey–Kramer multiple comparison test).

## Data Availability

The data presented in this study are available on request from the corresponding author.

## Supplementary Materials

The following supporting information can be downloaded at https://www.mdpi.com/article/10.3390/nu16244262/s1, Supplementary Table S1. YCFA medium composition; Supplementary Table S2A. Spearman correlation between gas production and top 30 bacterial genera; Supplementary Table S2B. Percentage of g_*Clostridium* population for each subject after 24 h of fermentation.
